# A randomized controlled trial comparing two ways of providing evidence-based drug information to GPs

**DOI:** 10.3109/02813432.2012.757071

**Published:** 2013-06

**Authors:** Ingmarie Skoglund, Cecilia Björkelund, Max Petzold, Ronny Gunnarsson, Margareta Möller

**Affiliations:** ^1^Department of Public Health and Community Medicine/Primary Health Care, Institute of Medicine, the Sahlgrenska Academy, University of Gothenburg, Sweden; ^2^The Research and Development Unit, Primary Health Care and Dental Care, Southern Älvsborg County, Region Västra Götaland1, Sweden; ^3^Institute of Medicine, the Sahlgrenska Academy, University of Gothenburg, Sweden; ^4^Centre for Health Care Sciences, Örebro County Council and School of Health and Medical Sciences, Örebro University, Sweden; ^5^Cairns Clinical School, School of Medicine and Dentistry, James Cook University, Australia

**Keywords:** Benefit aspects, drug information services, drug prescriptions, evidence-based medicine, general practice, general practitioner, motivational interviewing, primary health care, Sweden

## Abstract

**Objective:**

To investigate whether tailored evidence-based drug information (EBDI) to general practitioners (GPs) can change the proportion of ACE inhibitor prescriptions more effectively than EBDI provided as usual three and six months after the intervention.

**Design:**

Randomized controlled trial.

**Setting:**

GPs in southern Sweden working at primary health care centres (PHCCs) in seven drug and therapeutic committee areas.

**Intervention:**

EBDI tailored to motivational interviewing (MI) technique and focused on the benefit aspect was compared with EBDI provided as usual.

**Subjects:**

There were 408 GPs in the intervention group and 583 GPs in the control group.

**Main outcome measures:**

Change in proportion of ACE inhibitor prescriptions relative to the sum of ACE inhibitors and angiotensin receptor blockers, three and six months after the intervention.

**Results:**

The GPs’ average proportions of prescribed ACE inhibitors increased in both groups. No statistically significant differences in the change of proportions were found between intervention and control groups. Information was provided to 29% of GPs in both groups.

**Conclusion:**

This study could not prove that specially tailored EBDI using MI implements guidelines more effectively than EBDI provided as usual.

This study investigates whether the implementation of evidence-based drug information to general practitioners can be improved.

- There were no differences between drug information tailored with motivational interviewing focused on the benefit aspect and drug information provided as usual.
- The finding is in accordance with Cochrane reports on the use of motivational interviewing technique.
- Use of motivational interviewing in drug information has not previously been investigated in a randomized controlled trial.

## Introduction

Prescribed medication accounted for approximately 10% of resources used in Swedish health care in 2005 [1,2]. In view of steeply rising drug prices, various measures to limit prescription have been taken [1]. Swedish general practitioners (GPs) account for more than 50% of all drug prescriptions [3] indicating the importance of their knowledge in evidence-based medicine (EBM) [4]. The guideline on hypertension from the Swedish Council on Technology Assessment in Health Care (SBU) [5] is an EBM source.

The prevalence of hypertension has been estimated at 1.8 million (27%) Swedish adults; 60% with mildly, 30% moderately, and 10% severely elevated blood pressure [5]. In a study it was estimated that as many as 80% could be unsatisfactorily treated, entailing increasing risks [6]. New expensive drugs augment the discussion on cost-effectiveness [5,7]. The number of prescriptions of angiotensin II receptor blockers (ARB) increased in Sweden prior to studies of the effectiveness [8]. They were considered too expensive and without major benefits compared with angiotensin-converting enzyme (ACE) inhibitors [5]. A Danish study showed that there was no clear association between GPs’ clinical interest and their prescribing of new drugs such as ARB [9]. The Pharmaceutical Benefits Agency introduced limitations on ARB prescriptions with proposed savings of SKr 250 million (€28 million, $US 36 million) annually. The SBU's recommendations [5] for moderate hypertension (> 140/90) were; (a) encourage lifestyle changes, (b) prescribe low doses of one of the following drugs: thiazides, ACE inhibitors, calcium-blocking agents, and beta blockers; beta blockers were later downgraded to third-line treatment [10], (c) increase or add a low dose of the other drugs until acceptable blood pressure was attained, (d) ARB should only be used as a last-line drug.

Most drug information emanates from pharmaceutical companies [11] and is often too abundant [12,13]. In Sweden, evidence-based drug information (EBDI) to GPs is frequently provided by non-commercial medical information officers (MIOs) from the drug and therapeutic committees (DTCs) [14], funded by the county councils.

A Welsh qualitative study highlighted the complexity in drug prescribing [15]. Not one method [16] but a combination is preferable to modify prescribing behaviour [17]. A recent Swedish randomized controlled trial (RCT) among elderly patients showed no improvements in the patients’ quality of life when a prescription review was sent to the physician or to the physicians and to the patients themselves, compared with a control group [18]. According to a health technology assessment report [19] dissemination of printed educational materials, audit with feedback, and multifaceted interventions with educational outreach improve physician performance by 6–8% whereas reminders have twice the impact.

In a focus-group study, GPs’ thoughts on EBM and drug prescribing were related to benefit and results [20]. The core category was found to be: prompt and pragmatic benefit, delivered immediately, useful, and handy.

Motivational interviewing (MI) [21] is a change-oriented, client-centred, and governing methodology mainly used in the area of lifestyle change. Interest in the use of MI in Swedish health care is increasing [22].

The aim of this study was to investigate whether tailored EBDI using MI, based on previous findings on GPs’ thoughts on prompt and pragmatic benefit, can change GPs’ prescribing pattern of ACE inhibitors more effectively than EBDI provided as usual.

## Material and methods

An enquiry regarding participation was sent to all 29 Swedish DTCs; seven chose to participate. The MIOs of the seven DTCs, seven men and seven women (three GPs, 11 pharmacists), were previously assigned to provide information to GPs at specified PHCCs in the participating DTCs. The officers were matched pairwise as far as possible based on profession, number of GPs in their district, and sex; male and female pharmacists with an equal number of GPs in their domain, a female pharmacist officer with a female GP officer, and a male GP officer to a female GP officer. They were then randomized by an independent person. The GPs, the study objects, were as a result “cluster randomized” with their officer. There were 408 GPs in the intervention group and 583 in the control group.

Four male and three female officers (one GP, six pharmacists) provided tailored EBDI by using a motivational interviewing technique whereas three male and four female officers (two GPs and five pharmacists) provided EBDI as usual. In two DTCs the randomization resulted in control and intervention officers in the same DTC but never at the same PHCC.

The officers gathered in October 2004 and were lectured on the guideline on hypertension. Those randomized to provide benefit-tailored information [20] by using MI [21] were further trained for eight hours. The point of departure for the training was the individual GP's own thoughts and beliefs and the benefit aspects were emphasized. The method resembles that of patient-centred communication [23]. The MI training included role playing, which was videotaped [21], and the officers were given feedback. All officers were invited to cooperate within their own officer group. They were aware that a difference between the groups existed but did not know what constituted the difference. No one in either group had experienced a motivational interviewing technique before the study.

In data compilation we found 1031 physicians working at the PHCCs before and after the intervention. The majority were temporary doctors and substitutes. Statistics were calculated on all the physicians who worked at the PHCCs and who prescribed anti-hypertensive drugs during the study period. All physicians are referred to as GPs.

The intervention took place in November 2004. All GPs present at the 66 participating PHCCs, 28 in the intervention group and 38 in the control group, were presented with the new guidelines during a two hour session. The intervention group received EBDI using MI and tailored to GPs’ thoughts on prompt and pragmatic benefit, while the control group received EBDI as usual.

Prescription data from participating PHCCs for all antihypertensive drugs were collected from their computerized medical records according to the Anatomical Therapeutic Chemical Classification. The software systems for computerized medical records were Profdoc^®^, Medidoc^®^, and Swedestar^®^. Change in prescription from baseline (0–3 months before intervention) to the time periods 0–3 months and 4–6 months after the intervention were analysed. A separate file enabled linkage between data on prescription data and the prescriber.

The primary outcome was changed in the proportion of ACE inhibitor prescriptions relative to the sum of ACE inhibitors and ARB, comparing intervention and control groups during the two periods after the intervention.

A sample size calculation indicated that we needed an estimated total of 460 GPs (p < 0.05, power 90%); 991 were analysed. Statistics were calculated on the level of GPs.

Data were collected on several levels: (1) GPs’ prescriptions of antihypertensive drugs, (2) GPs, (3) PHCCs, and (4) geographical area including several PHCCs. Prescriptions were aggregated to produce just one change in proportion for each GP. A multilevel model was used to examine the effect of the levels *PHCC* and *geographical area* on the change in proportion of GPs’ ACE inhibitor prescriptions. As these levels explained less than 1% of the variation in the dependent variable/change in proportion of ACE inhibitor prescriptions, we decided to use the simpler multiple linear regression to compare the groups. The dependent variable was the change in GPs’ prescription proportion while independent variables were group allocation and those variables where groups differed at baseline: patients’ sex and type of clinic. The analysis was performed by intention-to-treat and per protocol.

The multilevel modelLing was made in the statistical program STATA. The multiple linear regression analyses were made in Epi-info 3.4.3 (CDC, Atlanta, USA).

## Results

**Figure 1.**
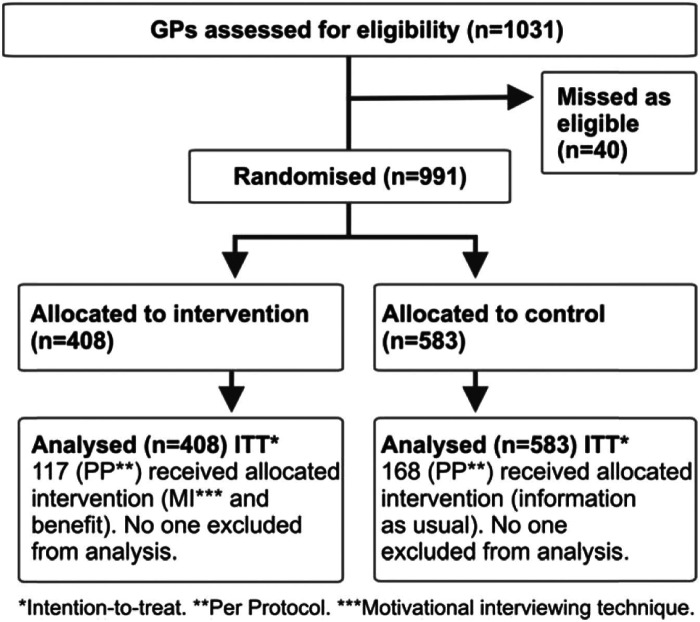
Flow of participating GPs through the study.

Of the 1031 GPs, 40 were not present at the time of study. Thus 408 GPs were allocated to intervention and 583 to control by randomization (Figure 1). At baseline there were more GPs working at private clinics in the intervention group and the average proportion of female patients was higher in the control group (Table I).

**Table I.** Baseline characteristics of 991 GPs in the intervention and control groups at baseline.

	Intervention group (n = 408)	Control group (n = 583)	Difference between groups (p-value)
Medical information officers^1^	6 pharmacists, 1 GP 4 males, 3 females	5 pharmacists, 2 GPs 3 males, 4 females	–
Primary healthcare centres	28	38	–
GPs’ age; years^2^	46 (11)	47 (11)	0.19
GPs’ sex; male/female^3^	248/153	328/250	0.13
Number of GPs working at private/public clinic^3^	31/377	0/583	**< 10^26^**
Proportion of GPs receiving allocated treatment^3^	29%	29%	0.96
Average proportion of female patients among GPs’ patients^2^	0.54 (0.19)	0.57 (0.20)	**0.016**
Average age of GPs’ patients; years^1^	69 (6.5)	68 (6.4)	0.13

Notes: ^1^Description of medical information officers’ profession (first line) and sex (second line). ^2^Mean (standard deviation). Difference between groups analysed with Student's t-test. ^3^Difference between groups analysed with chi-squared with Yates correction. Significant differences are shown in bold.

The proportion of ACE inhibitor prescriptions (average proportion for GPs) was increased in both groups at the three- and six-month follow-up. There were no significant differences in the change in prescription proportion between groups either with intention-to-treat (Table II) or per protocol analysis (Table III). Some 29% of the GPs received allocated information both in the intervention (117/408) and in the control group (168/583).

**Table II.** Proportion of ACE inhibitors prescribed by all GPs and change in this proportion over time (intention-to-treat).

	Intervention group	Control group
	n = 408	n = 583
Proportion of ACE inhibitors at 3 months before intervention, baseline^1, 2^	0.64 (0.26) 0.67 (0.50–0.83)	0.63 (0.28) 0.65 (0.45–0.85)
Relative change in proportion of ACE inhibitors 0–3 months after intervention^2,3^	+ 0.12 (0.43) + 0.029 (–0.11–0.32)	+ 0.12 (0.59) ± 0.00 (–0.17–0.27)
Relative change in proportion of ACE inhibitors 4–6 months after intervention^2,3^	+ 0.12 (0.47) + 0.051 (–0.13–0.25)	+ 0.13 (0.56) + 0.0040 (–0.14–0.26)

Notes: ^1^Proportion = number of ACE inhibitors prescribed divided by the sum of ACE inhibitors and ARBs. ^2^Upper line means (standard deviation). Lower line median (interquartile range). ^3^Relative change in proportion = Change in proportion of ACE inhibitors at follow-up divided by baseline proportion.

**Table III.** Change in proportion of ACE inhibitors prescribed by GPs actually receiving assigned intervention over time (per protocol).

	Intervention group	Control group
	n = 117	n = 168
Relative change in proportion of ACE inhibitors 0–3 months after intervention^1,2,3^	+ 0.14 (0.41) + 0.024 (–0.11–0.30)	+ 0.11 (0.49) + 0.053 (–0.17–0.25)
Relative change in proportion of ACE inhibitors 4–6 months after intervention^1,2,3^	+ 0.11 (0.47) + 0.049 (–0.13–0.24)	+ 0.14 (0.48) + 0.031 (–0.12–0.32)

Notes: ^1^Proportion = number of ACE inhibitors prescribed divided by the sum of ACE inhibitors and ARB. ^2^Upper line means (standard deviation). Lower line median (interquartile range). ^3^Relative change in proportion = change in proportion of ACE inhibitors at follow up divided by baseline proportion.

## Discussion

In this RCT we have investigated whether EBDI tailored with MI and focused on the benefit aspect implements guidelines to GPs more effectively than EBDI provided as usual. The same relative increase in ACE inhibitor prescriptions was found in both groups during the two periods 0–3 and 4–6 months after the intervention.

One of the strengths of the study is that a high percentage % (94; 62 out of 66) of the PHCCs completed the study by submitting data. Another strength was that the proportion of female GPs in our study (41%) was similar to that among physicians in Sweden 2004–2005 (38%) [24]. Further, 4.2 million of 9.1 million Swedish inhabitants live in the geographical area of the study representing large and medium-sized cities as well as rural areas.

Finally the MIOs, while aware that there was a difference between the groups, were not aware of what constituted the difference.

The limitations of our study are several: seven of the 29 DTCs took part; the others were occupied with other projects, or lacked time or employees to participate. The possibility of selection bias cannot be ignored. It was not possible for us to have control of how the information was provided to the GPs and the time for the tailored education might have been insufficient. Statistics were calculated on all the physicians who worked at the PHCCs and who prescribed anti-hypertensive drugs during the study period. We do not know to what extent those not present during the information-giving were reached by the information provided.

All prescriptions, both ongoing drug treatment prescribed by telephone and at a GP visit and those just initiated, were analysed. This might dilute the effect of change as is described in a North American mini-review [25]. The outcome was dependent on how the message on the use of ARBs was received. The other messages, except for the changed status of beta-blockers [10], were well known. The ARB message might have had a stronger impact if there were less competition in time use [25]. However, the issue of how to prescribe ARBs was well known beforehand by Swedish GPs from the medical debate.

The relative increase in the number of prescribed ACE inhibitors is in line with the recommendations from the SBU [5]. Because the numbers of prescribed ARB and ACE inhibitors has increased continuously since the year 2000 [8], it cannot be determined whether the increase in the number of ACE inhibitors prescriptions can be attributed to our intervention or not. Possible interpretations of the study results could either be equal impact of the information in both groups, no impact of the information in either group, or a combination of both explanations.

Motivational interviewing is described in the literature as an evidence-based method for lifestyle change, especially with regard to alcohol consumption [26,27]. In a review of RCTs on substance abuse, including alcohol, [28] MI as a brief individual intervention (1–4 sessions) significantly reduced abuse compared with no intervention; no significant differences were seen compared with treatment as usual. A meta-analysis of RCTs on MI compared with treatment as usual mainly in primary health care [29] showed significant effects on body mass index, systolic hypertension, total blood cholesterol, and alcohol measurement in about 75% of patients. However, the “as usual” in this study meant a GP-centred approach. This is not regarded as the “gold standard” in contemporary patient consultations [30]. Patient education using MI on diabetes by nurses [22] rendered no improvements on HbA1c compared with education as usual.

One major difference between our study and others is that GPs were informed in a group instead of individually. Another difference is that GPs differ from patients, having a different pre-understanding of the context than patients. In this study, the GPs were informed in a group and had a different relationship to the information provider than patients have to a care provider. However, an interesting similarity is that as in the review on MI and substance abuse [28] no differences were seen between MI and treatment as usual. A plausible explanation of the results in our study is that MI and other interventions share non-specific therapeutic factors, such as attention and therapeutic alliance, which might contribute to 30% of the effect [31].

## Conclusion

This study could not prove that specially tailored EBDI using MI to GPs changes the outcome more than EBDI provided as usual.

## References

[1] The National Board of Health and Welfare (2007). Läkemedelsförsäljningen i Sverige – analys och prognos [Pharmaceutical sales in Sweden – analysis and forecast] [homepage on the Internet]. http://www.socialstyrelsen.se/Lists/Artikelkatalog/Attachments/8952/2007-103-5_20071035.pdf.

[2] Ministry of Health and Social Affairs (2007). Health and medical care in Sweden [homepage on the Internet]. Government offices of Sweden.

[3] ABLA II (2001). Mindre sjukdom och bättre hälsa genom ökad följsamhet till läkemedelsordinationerna [Less disease and better health through higher adherence to drug prescriptions. The role of the health professionals].

[4] Sackett DL, Rosenberg WM, Gray JA, Haynes RB, Richardson WS (1996). Evidence based medicine: What it is and what it isn't. BMJ.

[5] SBU (2004). Moderately elevated blood pressure: A systematic review [in Swedish].

[6] Barth T (2006). Näsholm CMotiverande Samtal – MI. Att hjälpa en människa till förändring på hennes egna villkor [Motivational interviewing – MI. Helping a person to change on her own terms].

[7] TLV (2008). Högt blodtryck. Slutrapport. En genomgång av de läkemedel som sänker blodtrycket [Hypertension. Final report. A review of the drugs that decrease blood pressure] [homepage on the Internet].

[8] Wettermark B, Angman A, Hjemdahl P (2009). [Fully possible to reduce the costs of hypertension treatment]. Lakartidningen.

[9] Dybdahl T, Sondergaard J, Kragstrup J, Kristiansen IS, Andersen M (2011). Primary care physicians’ adoption of new drugs is not associated with their clinical interests: A pharmacoepidemiologic study. Scand J Prim Health Care.

[10] Carlberg B, Samuelsson O, Lindholm LH (2004). Atenolol in hypertension: is it a wise choice?. Lancet.

[11] Tobin L, de Almedia Neto AC, Wutzke S, Patterson C, Mackson J, Weekes L (2008). Influences on the prescribing of new drugs. Aust Fam Physician.

[12] Caamano F, Figueiras A, Gestal-Otero JJ (2002). Influence of commercial information on prescription quantity in primary care. Eur J Public Health.

[13] Skoglund I, Bjorkelund C, Mehlig K, Gunnarsson R, Moller M (2011). GPs’ opinions of public and industrial information regarding drugs: A cross-sectional study. BMC Health Serv Res.

[14] Sjöqvist F (2002). Drug and therapeutic committees: A Swedish experience [homepage on the Internet].

[15] Butler CC, Rollnick S, Pill R, Maggs-Rapport F, Stott N (1998). Understanding the culture of prescribing: Qualitative study of general practitioners’ and patients’ perceptions of antibiotics for sore throats. BMJ.

[16] Oxman AD, Thomson MA, Davis DA, Haynes RB (1995). No magic bullets: A systematic review of 102 trials of interventions to improve professional practice. CMAJ.

[17] O’Brien MA, Rogers S, Jamtvedt G, Oxman AD, Odgaard-Jensen J, Kristoffersen DT (2007). Educational outreach visits: Effects on professional practice and health care outcomes. Cochrane Database Syst Rev.

[18] Olsson IN, Runnamo R, Engfeldt P (2012). Drug treatment in the elderly: An intervention in primary care to enhance prescription quality and quality of life. Scand J Prim Health Care.

[19] Grimshaw JM, Thomas RE, MacLennan G, Fraser C, Ramsay CR, Vale L (2004). Effectiveness and efficiency of guideline dissemination and implementation strategies. Health Technol Assess.

[20] Skoglund I, Segesten K, Bjorkelund C (2007). GPs’ thoughts on prescribing medication and evidence-based knowledge: The benefit aspect is a strong motivator. A descriptive focus group study. Scand J Prim Health Care.

[21] Miller WR, Rollnick S (2002). Motivational interviewing: Preparing people for change.

[22] SBU (2009). Patientutbildning vid diabetes – en systematisk litteraturöversikt [Patient education in diabetes – a systematic literature review].

[23] Elwyn G, Edwards A, Wensing M, Hood K, Atwell C, Grol R (2003). Shared decision making: Developing the OPTION scale for measuring patient involvement. Qual Saf Health Care.

[24] Svenska Läkarförbundet [Swedish Medical Association] Arbetsmarknad Läkarfakta [Labour market. Facts about physicians] [homepage on the Internet]. 2007.

[25] Maclure M, Nguyen A, Carney G, Dormuth C, Roelants H, Ho K (2006). Measuring prescribing improvements in pragmatic trials of educational tools for general practitioners. Basic Clin Pharmacol Toxicol.

[26] Miller WR, Rollnick S (2010). Motiverande samtal. Att hjälpa människor till förändring [Motivational Interviewing. Helping people to change].

[27] Hettema J, Steele J, Miller WR (2005). Motivational interviewing. Annu Rev Clin Psychol.

[28] Smedslund G, Berg RC, Hammerstrom KT, Steiro A, Leiknes KA, Dahl HM (2011). Motivational interviewing for substance abuse. Cochrane Database Syst Rev.

[29] Rubak S, Sandbaek A, Lauritzen T, Christensen B (2005). Motivational interviewing: A systematic review and meta-analysis. Br J Gen Pract.

[30] Stewart M, Brown JB, Donner A, McWhinney IR, Oates J, Weston WW (2000). The impact of patient-centered care on outcomes. J Fam Pract.

[31] Lambert M (1986). Implications of psychotherapy outcome research for eclectic psychotherapy: Brunner Mazel.

